# Endoscopic Resection of a Cavernous Hemangioma in the Sigmoid Colon: A Case Report

**DOI:** 10.1016/j.gastha.2023.12.008

**Published:** 2023-12-25

**Authors:** Noora Al-Khater, Mohamed Mohamed, Afra Juma, Faisal Abubaker, Sameer Ansari

**Affiliations:** 1 Internal Medicine Department, King Hamad University Hospital, Busaiteen, Kingdom of Bahrain; 2 Gastroenterology and Hepatology Department, King Hamad University Hospital, Busaiteen, Kingdom of Bahrain; 3 Pathology Department, King Hamad University Hospital, Busaiteen, Kingdom of Bahrain

**Keywords:** Hemangioma, Sigmoid Colon, Abdominal Pain, Endoscopy

## Abstract

Hemangiomas in the gastrointestinal tract are extremely rare, benign vascular tumors, known for their associated complication of bleeding. They are usually difficult to diagnose, despite the characteristic definition of radiolucent phleboliths on radiology and purplish, tannish brown nodule or polyp on endoscopy. Surgical resection is the treatment of choice. We describe a rare case of sigmoid colon cavernous hemangioma in a 49-year-old male who underwent colonoscopy for lower abdominal pain and revealed a large pedunculated polyp in the sigmoid colon measuring 1.7 cm in diameter. The hemangioma was completely resected endoscopically via hot snare with a favorable outcome.

## Introduction

Lower gastrointestinal tract hemangiomas are rare benign vascular tumors, most located in the rectosigmoid region.^1^ Colonic hemangiomas are important as they can cause massive hemorrhage, intestinal obstruction, intussusception, or volvulus.^1^^,^^2^ Diagnosis is usually confirmed by macroscopic evaluation during endoscopy, as histopathological diagnosis is difficult due to the high risk of bleeding associated with manipulation during biopsy.^2^ Operative resection is the recommended method of treatment, but endoscopic treatment with close follow-up is possible with polypoid tumors with a narrow base.^1^

## Case Report

We report a 49-year-old male with a medical history of hypertension and obesity and a past surgical history of cholecystectomy. He was reviewed in gastroenterology clinic with complaints of left-sided lower abdominal pain, associated with constipation. He had no bleeding per rectum or weight loss. A blood test revealed no evidence of anemia. A computed tomography scan of the abdomen was done, which showed no evidence of colonic disease.

**Figure 1:**
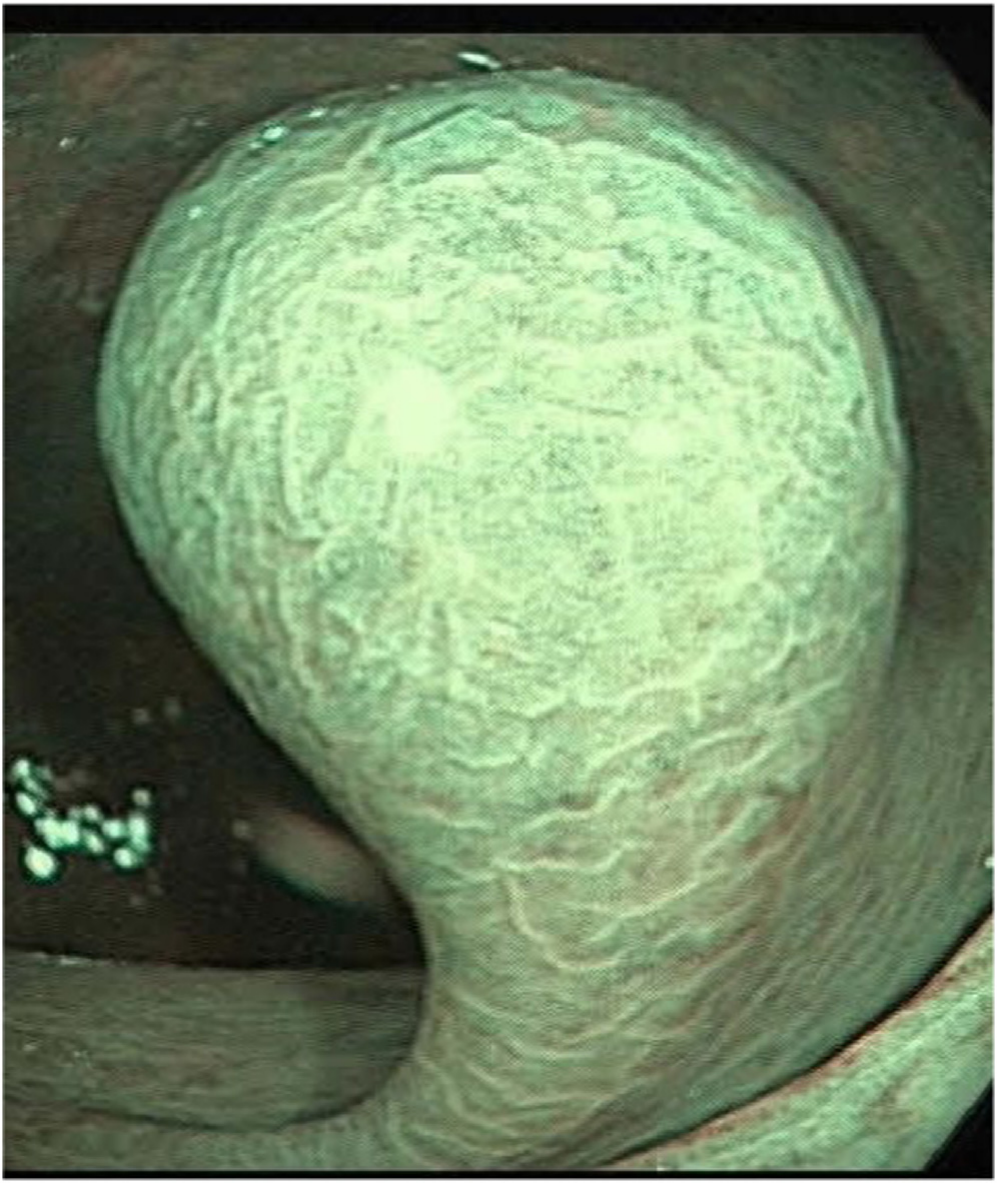
Image taken during colonoscopy showing a 17-mm pedunculated polyp seen in the sigmoid colon.

Colonoscopy was performed and showed at the level of the sigmoid colon, a large 17-mm pedunculated polyp (Figure 1).

**Figure 2:**
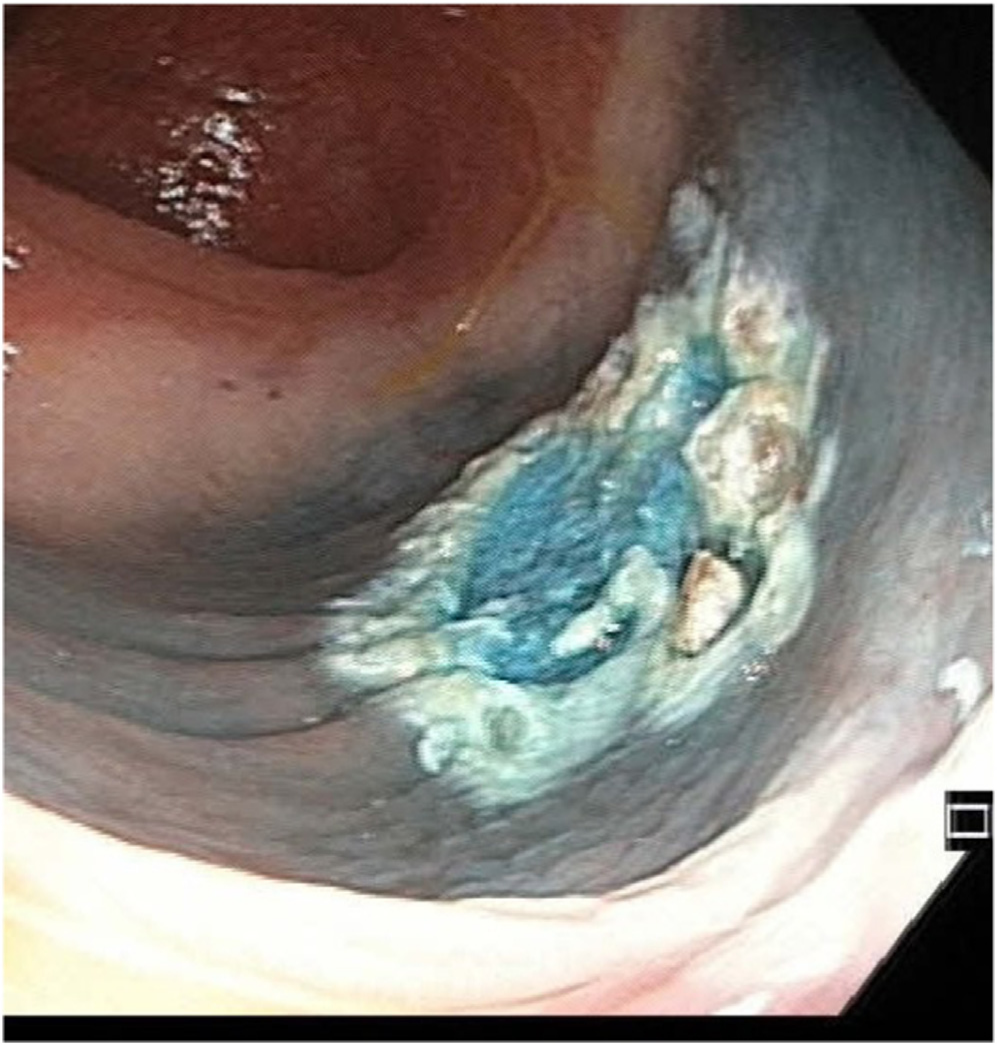
Image taken showing complete resection of the polyp after snare-tip soft coagulation of the margins.

A large, pedunculated polyp with a broad stalk and an indistinct, cloud-like surface was found in the sigmoid during colonoscopy. The polyp showed a lifting sign after injection of 4 mL of a mixture of methylene blue and normal saline. Using Olympus CF HQ 190 ERBE Vio 2 endocut Q (effect 3, duration 1, interval 6), the polyp was resected completely (Figure 2). Two clips were then applied at the defect site after snare-tip soft coagulation of the margins.

The patient was admitted to the day-case unit for observation after the procedure and was discharged in stable condition. After the procedure, the patient did not develop any bleeding.

**Figure 3:**
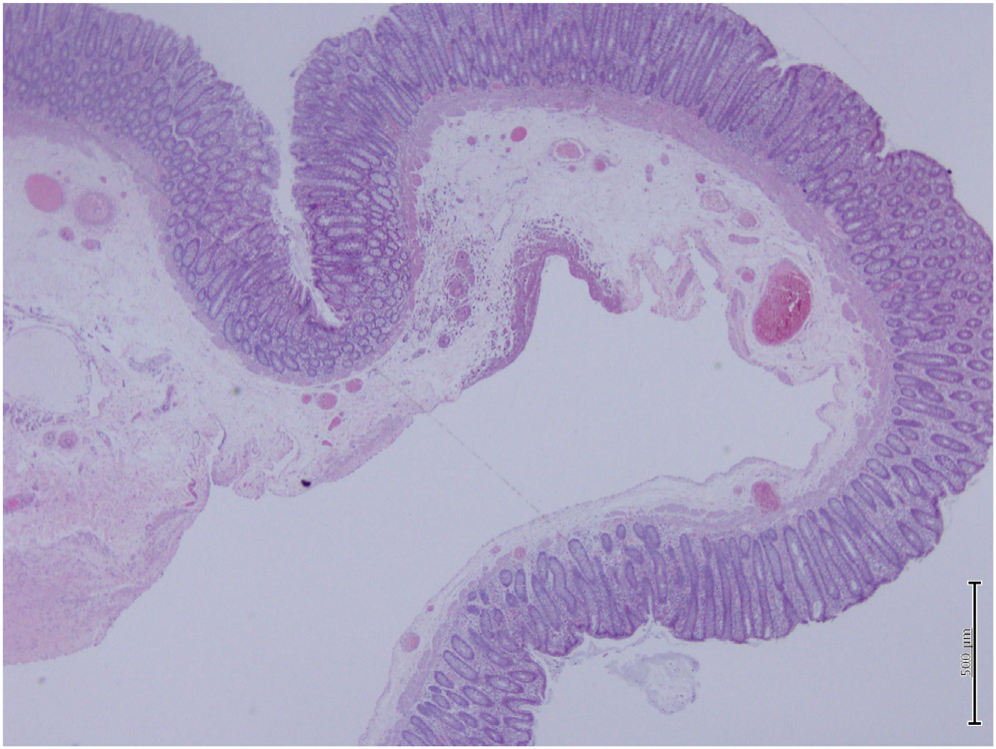
Microscopic examination showing a polyp formed by a localized, relatively well-outlined, sinus-like submucosal hemangioma with unremarkable overlying mucosal.

Gross examination revealed a polypoidal tissue, tannish white in color and soft in consistency with the core of polyp showing congestion. Microscopic examination revealed a polyp formed by a localized, relatively well-outlined, sinus-like submucosal hemangioma. The overlying mucosa is unremarkable. (Figure 3).

The patient underwent a follow-up colonoscopy after 6 months, which showed no recurrence.

## Discussion

Hemangiomas in general are benign vascular tumors seen anywhere in the body, usually present at birth and the majority undergo spontaneous regression.^3^ Hemangioma in the gastrointestinal tract is rare, with the colonic segment being the second most common site of gastrointestinal hemangiomas.^4^^,^^5^ Within the colon, the rectosigmoid is the most commonly affected area, as was found in our case.^5^

Hemangiomas usually present as intraluminal polypoidal growth or masses, but they can have intramural extension by infiltrating the submucosa and muscularis, making them difficult to excise.^4^^,^^6^ About 80% of colonic hemangiomas are cavernous hemangiomas, whereas capillary hemangiomas are usually found in less than 10% of the cases.^4^^,^^6^

Majority of patients exhibit symptoms of intraluminal bleeding, which is usually painless and can sometimes show general weakness due to iron-deficient anemia.^5^ Hemangiomas can also contain phleboliths, which are calcified well-circumscribed densities seen in around 50% of cases.^2^ Although infrequent, they can also be a cause of obstruction. The colonic hemangiomas can act as a lead point for intussusception, resulting in obstruction, or they can grow diffusely along the wall occluding the lumen.^2^ Patients can also present with abdominal pain and tenesmus.^6^ Gastrointestinal hemangiomas most commonly affects young men.^5^ Our patient has similar demographics being a 49-year-old male.

The treatment of choice for large diffuse lesions is surgical resection. However, previous studies have demonstrated the use of endoscopic mucosal resection and endoscopic submucosal dissection in the resection of a polypoidal and a pedunculated hemangioma, respectively^7^^,^^8^

In addition, in the presence of a pedunculated lesion, endoscopic treatment with hot snare polypectomy becomes a viable less invasive approach, as was reported in 2 different case reports.^2^^,^^4^ No bleeding or perforation occurred in these cases, as in our patient as well.

A surveillance colonoscopy was done, which showed treatment success and confirmed no recurrence. In conclusion, we believe that in such cases, endoscopic polypectomy might be a safe therapeutic option for patients with pedunculated cavernous hemangiomas. However, more studies are required to validate this treatment modality and its efficacy.
